# Estimation of causal effect measures with the R-package stdReg

**DOI:** 10.1007/s10654-018-0375-y

**Published:** 2018-03-14

**Authors:** Arvid Sjölander

**Affiliations:** Karolinska Institute, Nobels väg 12 A, 171 77 Stockholm, Sweden

**Keywords:** Attributable fraction, Causal effect, Cox proportional hazards regression, Logistic regression, Number needed to treat, Relative excess risk due to interaction

## Abstract

Measures of causal effects play a central role in epidemiology. A wide range of measures exist, which are designed to give relevant answers to substantive epidemiological research questions. However, due to mathematical convenience and software limitations most studies only report odds ratios for binary outcomes and hazard ratios for time-to-event outcomes. In this paper we show how logistic regression models and Cox proportional hazards regression models can be used to estimate a wide range of causal effect measures, with the R-package stdReg. For illustration we focus on the attributable fraction, the number needed to treat and the relative excess risk due to interaction. We use two publicly available data sets, so that the reader can easily replicate and elaborate on the analyses. The first dataset includes information on 487 births among 188 women, and the second dataset includes information on 2982 women diagnosed with primary breast cancer.

## Introduction

A common aim of epidemiologic research is to estimate the causal effect of an exposure on an outcome. To control for potential confounders it is common to use logistic regression models for binary outcomes and Cox proportional hazards (PH) regression models for time-to-event outcomes. Methods for fitting these models are implemented in all major statistical software, which makes them easily accessible to applied epidemiologists.

Logistic regression models and Cox PH regression models are parametrized in terms of log odds ratios and log hazard ratios, respectively. These parameters are mathematically convenient since they are unrestricted, i.e. they can have values anywhere in the range ($-\,\infty ,\infty$). Thus, logistic regression models and Cox PH regression models will never produce parameter estimates that are outside the supported range. Arguably though, the log odds ratio and the hazard ratio are usually not the most intuitive or relevant measures of the exposure effect. Both are often misinterpreted, in particular among applied epidemiologists and clinicians without statistical training [1–3], and neither is directly informative about the public health impact of the exposure, since they do not take the exposure prevalence into account. Furthermore, when assessing interactions between two exposures, it has been argued that the risk differences are more appropriate than odds ratios or hazard ratios [4].

There exist many other suggestions for causal effect measures in the literature, which are supposed give more relevant answers to substantive epidemiological research questions [5]. For instance, the risk difference and the survival difference are relatively easy to interpret and communicate to non-statisticians. The attributable fraction (AF) and the number needed to treat (NNT) are directly informative about the public health impact of the exposure/treatment. The relative excess risk due to interaction (RERI), the synergy index (S) and the attributable proportion due to interaction (AP) measure the amount of interaction between two exposures on the additive scale. Methods have been developed to estimate these (and related) measures from logistic regression models and Cox PH regression models (see Rothman et al. [5] and the references therein), and several of these methods have been implemented in statistical software. However, these implementations are typically scattered across various packages and commands, with diverse syntax and functionality.

The aim of this paper is to show how one single R-package, stdReg [6], can be used to estimate a wide range of causal effect measures, including all those mentioned above. Briefly, the package uses ‘regression standardization’ to estimate standardized probabilities from a fitted regression model. We described this procedure in a recent paper [7]. In the current paper we show how the standardized probabilities can subsequently be contrasted to form various measures of the exposure effect. A few simple effect measures are already implemented in the stdReg package, such as the risk difference and the risk ratio. However, due to the wide range of existing measures, and the creativity among epidemiologists to invent new measures, it would be virtually impossible to implement them all. Rather, we show in this paper how an analyst may obtain, with a minimal amount of programming, a desired effect measure from the standardized probabilities estimated by stdReg. We also show how the delta method can be used to estimate the variance and construct confidence intervals for the desired effect measure.

To our knowledge, there are currently only two packages in R that carry out regression standardization; stdReg and margins. However, margins is restricted to linear effects (e.g. differences) and cannot be used to compute other measures of causal effects. Furthermore, margins is restricted to generalized linear models and does not support models for time-to-event data.

The paper is organized as follows. In “Regression standardization” section we briefly review the method of regression standardization; we refer to Sjölander [7] for a more detailed account. In the subsequent sections we show how the stdReg package can be used to estimate various effect measures. For illustration we focus on the AF (“The AF” section), the NNT (“The NNT” section) and the RERI (“The RERI” section). We use two publically available datasets, so that the reader can easily replicate and elaborate on the analyses. The first dataset includes information on 487 births among 188 women, and the second dataset includes information on 2982 women diagnosed with primary breast cancer. These datasets are borrowed from the AF package [8]; the help files for this package provide a thorough description of the data. We assume that the reader has some experience with R programming, and with the glm function from the stats package and the coxph function from the survival package.

## Regression standardization

Let *X* and *Y* be the exposure and outcome of interest, respectively. For the moment we assume that the outcome is binary (0/1), but we do not make any particular assumption about the exposure. Let $Y_x$ be the potential outcome [9, 10] for a given subject, if that subject would be exposed to the fixed level $X=x$. Finally, let $p(Y_x=1)$ be the counterfactual probability of the outcome if all subjects in the population would hypothetically be exposed to $X=x$. We here use the term ‘population’ in the usual epidemiological sense, i.e. as referring to a hypothetical, infinitely large superpopulation, from which the observed sample was drawn [5].

Counterfactual probabilities are cornerstones in the modern theory of causal inference, and can be used to define a wide range of effect measures. For instance, when the exposure is binary the causal risk difference and risk ratio are defined as $p(Y_1=1)-p(Y_0=1)$ and $p(Y_1=1)/p(Y_0=1)$, respectively. We will use counterfactual probabilities to define the AF (“The AF” section), the NNT (“The NNT” section) and the RERI (“The RERI” section).

To estimate $p(Y_x=1)$ without bias from observational (i.e. non-randomized) data, it is necessary to control for confounding. Let *Z* be a set of measured confounders and let *p*(*Y*|*X*, *Z*) be the conditional distribution of *Y*, given *X* and *Z*. If *Z* is sufficient for confounding control, then

1 $$\begin{aligned} p(Y_x=1)=E\{p(Y=1|X=x,Z)\}, \end{aligned}$$

where the expectation on the right-hand side is taken over the population distribution of *Z* [10]. Regression standardization attempts to estimate $p(Y_x=1)$ by estimating the right-hand side of (1), as follows. In a first step, a regression model for *p*(*Y*|*X*, *Z*) is fitted to the observed data, e.g. a logistic regression model. In a second step, the fitted model is used to estimate $p(Y=1|X=x,Z)$ for the fixed level $X=x$, and for each observed level of *Z* in the dataset. In a third step, these estimates are averaged. In concise notation we thus have that

2 $$\begin{aligned} {{\hat{p}}}(Y_x=1)=\frac{\sum _{i=1}^n{\hat{p}}(Y=1|X=x,Z_i)}{n}, \end{aligned}$$

where $Z_i$ is the observed level of *Z* for subject *i*, $i=1,\ldots ,n$, and ${\hat{p}}(Y=1|X=x,Z_i)$ is the estimate of $p(Y=1|X=x,Z_i)$ obtained from the fitted regression model.

Using the same fitted model from the first step, the second and third steps above are repeated for different values of *x*. Once the counterfactual probabilities $p(Y_x=1)$ have been estimated for different values of *x*, we may contrast these to obtain desired measures of the exposure effect. When *X* is categorical with few levels (e.g. binary), it is possible to estimate $p(Y_x=1)$ for all possible values of *x*. When *X* is multilevel categorical or continuous, one would typically have to focus on a few selected values of interest.

In many scenarios, the outcome is a time-to-event, e.g. time to death or time to relapse for cancer patients. To have a simple and uniform notation we then let *Y*(*t*) be the indicator of having the event before a fixed time-point *t*, e.g. 5 years from birth or from age at cancer diagnosis. Thus, $p\{Y_x(t)=1\}$ is the counterfactual probability of having the event before time *t* if all subjects in the population would hypothetically be exposed to $X=x$. With these definitions, regressions standardization proceeds as outlined above, for any fixed time-point *t*. However, when the underlying outcome is a time-to-event, it is more appropriate to use a Cox PH regression model than a logistic regression model. First, because the Cox PH regression model deals more naturally with censoring than the logistic regression model. Second, because in the analysis one may want to consider a range of different time-points. If a logistic regression model is used, then a new model has to be fitted for each time-point. In contrast, one single Cox PH regression model may be used to estimate $p\{Y(t)=1|X=x,Z\}$ for arbitrary values of *t*. For details on this estimation procedure we refer to Sjölander [7].

Typically, we want to have the variance of the estimated effect, so that we can, for instance, construct confidence intervals and hypothesis tests. The asymptotic variance can be obtained with the delta method [11], as follows. Let ${\mathbf{p}}$ be the vector of counterfactual probabilities and let $g({\mathbf{p}})$ be the desired effect measure. For instance, when *X* is binary and we wish to estimate the causal risk difference we have that ${\mathbf{p}}=\{p(Y_1=1),p(Y_0=1)\}$ and $g({\mathbf{p}})=p(Y_1=1)-p(Y_0=1)$. Let ${\hat{{\mathbf{p}}}}$ be the estimate of ${\mathbf{p}}$ and let $\text{var}({\hat{{\mathbf{p}}}})$ be the variance-covariance matrix for ${\hat{{\mathbf{p}}}}$. Let ${\widehat{\text{var}}}({\hat{{\mathbf{p}}}})$ be an estimate of $\text{var}({\hat{{\mathbf{p}}}})$. Using the delta method it can be shown that the estimated effect $g({\hat{{\mathbf{p}}}})$ has an asymptotic normal distribution, with variance equal to

3 $$\begin{aligned} \text{var}\{g({\hat{{\mathbf{p}}}})\}=\frac{\partial g({\mathbf{p}})}{\partial {\mathbf{p}}}\text{var}({\hat{{\mathbf{p}}}})\frac{\partial g({\mathbf{p}})}{\partial {\mathbf{p}}^T}. \end{aligned}$$

An estimate of the variance, ${\widehat{\text{var}}}\{g({\hat{{\mathbf{p}}}})\}$, is obtained by replacing ${\mathbf{p}}$ and $\text{var}({\hat{{\mathbf{p}}}})$ in (3) with ${\hat{{\mathbf{p}}}}$ and ${\widehat{\text{var}}}({\hat{{\mathbf{p}}}})$, respectively. The estimated variance can, for instance, be used to construct a standard Wald-type 95% confidence interval for $g({\mathbf{p}})$ on the form $g({\hat{{\mathbf{p}}}})\pm 1.96\sqrt{{\widehat{\text{var}}}\{g({\hat{{\mathbf{p}}}})\}}$. For parameters that are restricted to positive values, such as the NNT (“The NNT” section), it is desirable to ensure that the confidence interval is restricted to positive values as well. This may be accomplished by first computing the standard Wald confidence interval for the logarithm of the parameter, then back-transforming to the original scale, which gives the confidence interval $\text{exp}[\text{log}\{g({\hat{{\mathbf{p}}}})\}\pm 1.96\sqrt{{\widehat{\text{var}}}\{g({\hat{{\mathbf{p}}}}) \}}/g({\hat{{\mathbf{p}}}})]$. This transformed confidence interval typically has a coverage probability closer to the nominal level than the untransformed confidence interval.

We end this section by emphasizing that, in real observational studies, it would rarely be possible to measure all confounders for the exposure-outcome association. This means that it is rarely possible to estimate the counterfactual probabilities such as $p(Y_x=1)$ without bias. However, if the study is well designed, and potential confounders have been carefully selected, then the bias may be relatively small.

## The AF

### Definition

The AF measures the proportion of outcome events that would be prevented if the exposure was hypothetically eliminated from the population. For binary outcomes the AF is defined as

4 $$\begin{aligned} \text{AF}=1-\frac{p(Y_0=1)}{p(Y=1)}, \end{aligned}$$

see, for instance, Sjölander [12]. Here, $p(Y=1)$ is the factual probability (prevalence) of the outcome in the population of interest, and $p(Y_0=1)$ is the counterfactual probability of the outcome if the exposure was eliminated (set to 0). For instance, if $p(Y=1)=0.05$ and $p(Y_0=1)=0.01$, then an elimination of the exposure would prevent $1-0.01/0.05=80\%$ of all outcomes. We note that the definition in (4) does not assume that the exposure is binary per se, but it does assume that there is a natural ‘zero-level’, at which the exposure is completely absent.

For time-to-event outcomes, the AF is defined as in (4), but with *Y* and $Y_0$ replaced by *Y*(*t*) and $Y_0(t)$, respectively, for a given *t* [13, 14]. Thus, the AF measures the proportion of outcome events that would be prevented before time *t* if the exposure was eliminated at baseline ($t=0$). For many time-to-event outcomes, the AF is a decreasing function of *t*. For instance, if the outcome is death and *t* is 200 years from birth, then the AF is 0, since no realistic exposure intervention can prevent a subject from dying within 200 years from birth.

For details on model-based estimation of the AF we refer to Sturmans et al. [15], Deubner et al. [16], Greenland and Drescher [17], Chen et al. [13, 14], Sjölander and Vansteelandt [18, 19].

### Estimation with logistic regression models

We illustrate the methods with the dataset clslowbwt from the AF package. This dataset includes information on 487 births among 188 women. We will use the variables lbw (a binary indicator of whether the newborn child has low birthweight, defined as a birthweight smaller or equal to 2500 g), smoker (a binary indicator of whether the mother smoked during pregnancy), race (race of the mother, coded as 1. White, 2. Black or 3. Other), age (age of the mother), and id (a unique identification number for each mother). Our aim is to estimate the proportion of low birthweights that would be prevented if nobody would smoke during pregnancy. We will control for mother’s race and age in the analysis.

The first step is to fit a logistic regression model that relates the outcome (low birthweight) to the exposure (smoking) and measured confounders (race and age). This is done by

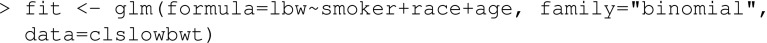



which stores the fitted model into an object called fit. The results are summarized, without ‘significance stars’, by

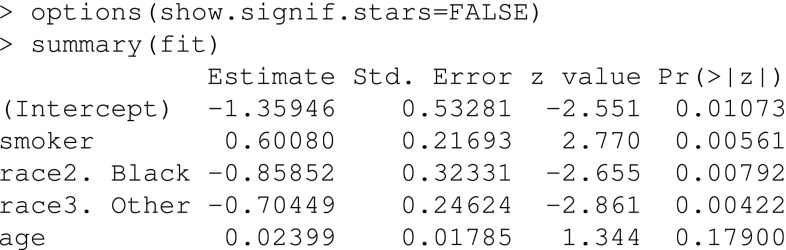



We observe that both smoking and race are significantly (at 5% significance level) associated with low birthweight, whereas age is not.

The next step is to estimate standardized probabilities. This is done with the stdGlm function in the stdReg package, by 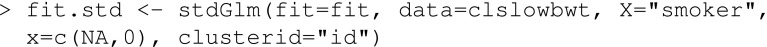


which stores the standardization results into an object called fit.std. The fit argument specifies a fitted generalized linear (e.g. logistic) model and the data argument specifies the data frame used to fit the model. The X argument specifies the name of the exposure variable and the x argument specifies fixed exposure levels for which we wish to estimate the counterfactual probability $p(Y_x=1)$. We here use a trick; by setting x to NA each subject retains her own factual exposure level, so that the factual outcome probability $p(Y=1)$ is estimated. This is useful, since the definition of AF in (4) involves $p(Y=1)$. By setting x to 0, the counterfactual probability $p(Y_0=1)$ is estimated. The argument clusterid specifies a variable that defines clusters in the data, e.g. mothers with multiple births. This has no effect on the estimates, but makes the stdGlm function use the ‘sandwich formula’ [20] to correct the variance of the estimates for within-cluster dependencies. Summarizing the results gives

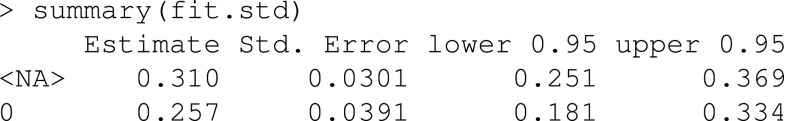



Thus, the factual probability of low birthweight is estimated to be 31.0%, and the counterfactual probability, had nobody smoked during pregnancy, is estimated to be 25.7%.

The fit.std object has (among other things) an element called est, which is a vector containing the estimated standardized probabilities in the order specified by the x argument, and an element called vcov, which is the (estimated) variance-covariance matrix of the estimates. We now define a function that uses est to estimate the AF:

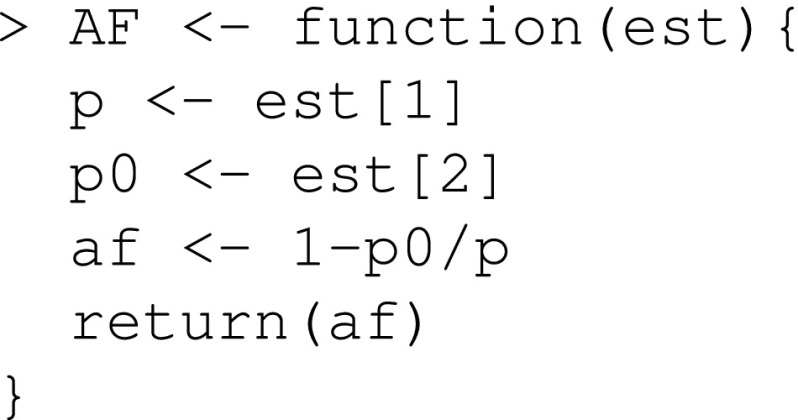



Using this function gives

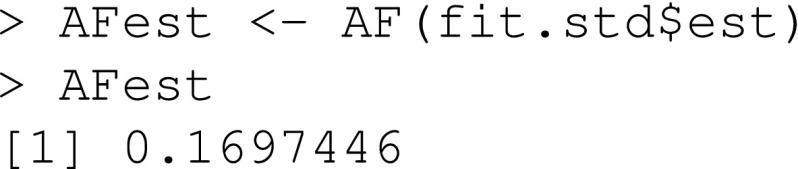



Hence, the analysis suggests that around 17% of all low birthweights would be prevented if nobody would smoke during pregnancy. We emphasize that this causal interpretation crucially hinges on race and age being sufficient for confounding control.

The stdReg package has a function confint, which uses the delta method to compute a Wald-type confidence interval for a parameter specified as a function of standardized probabilities. Using this function gives

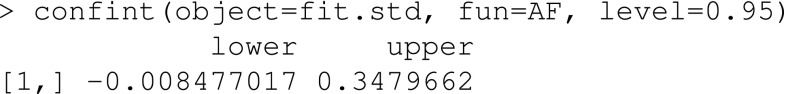



The optional argument level controls the coverage probability of the interval, and defaults to 0.95. The 95% confidence interval is quite wide, and suggests that the true AF may be as high as 34.8%. Furthermore, it includes the value 0, so at 5% significance level we cannot reject the null hypothesis that low birthweight is not prevented by eliminating smoking.

### Estimation with Cox PH regression models

We illustrate the methods with the dataset rott2 from the AF package. This dataset includes information on 2982 women diagnosed with primary breast cancer from the Rotterdam tumor bank in the Netherlands. Follow-up is measured in months since diagnosis, and ranges from 1 to 231 months. We will use the variables rf (follow-up time, measured in months, since diagnosis), rfi (an indicator of whether the patient died or had a relapse before censoring), chemo (an indicator of whether the patient received chemotherapy, coded as "yes" or "no"), age (patient’s age at surgery), meno (menopausal status, coded as 0 for pre and 1 for post), size (tumor size in three categories: "$\texttt{<=20mm}$", "$\texttt{>20-50mmm}$" and "$\texttt{>50mm}$"), grade (tumor grade; 2 or 3), nodes (the number of positive lymph nodes, ranging from 0 to 34), pr (progesterone receptors, fmol/l), and er (oestrogen receptors, fmol/l). Chemotherapy is supposed to give the patients a better prognosis, e.g. to prevent deaths and relapses. Our aim is to estimate the proportion of deaths and relapses that would be prevented if all patients received chemotherapy. We will control for age, menopausal status, tumor size, tumor grade, lymph nodes, progesterone and oestrogen receptors in the analysis.

To be consistent with the notation in “Definition” section, where we used values 0 and 1 for ‘unexposed’ and ‘exposed’, respectively, we first define the binary exposure variable

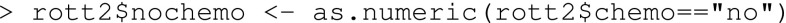



We fit a Cox PH regression model that relates the outcome (time to death/relapse) to the exposure (absence of chemotherapy) and measured confounders (age, menopausal status, tumor size, tumor grade, lymph nodes, progesterone and oestrogen receptors). This is done by

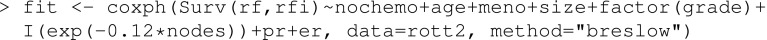



We here used the transformation $\text{exp}(-\,0.12*\texttt{nodes})$, since previous authors have shown that this gives a better model fit [21]. We obtain the results

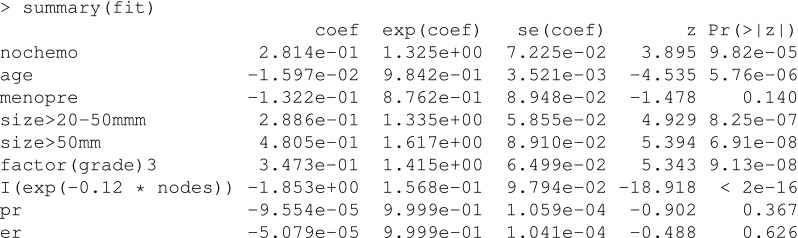



We observe that the absence of chemotherapy is indeed associated with a higher rate of death/relapse. We next use the fitted model to estimate standardized probabilities. This is done with the stdCoxph function in the stdReg package, by

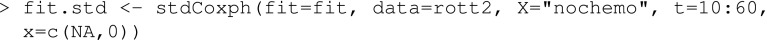



The syntax for stdCoxph is similar to the syntax for stdGlm. However, stdCoxph has an additional argument t, which specifies the time points at which to carry out the standardization; we here consider a sequence of 10 through 60 months after diagnosis. summary(fit.std) produces a long output displaying the results for each of these time points separately (not shown here).

Similar to stdGlm, the stdCoxph function creates an object with elements est and vcov. However, when created by the stdCoxph function, the element est is a matrix containing estimated standardized survival probabilities, $1-{\hat{p}}\{Y_x(t)=1\}$, for the specified values of t (in rows) and x (in columns). The element vcov is a list containing the variance–covariance matrices at each value of t. We now define a function that uses est to estimate the AF: 
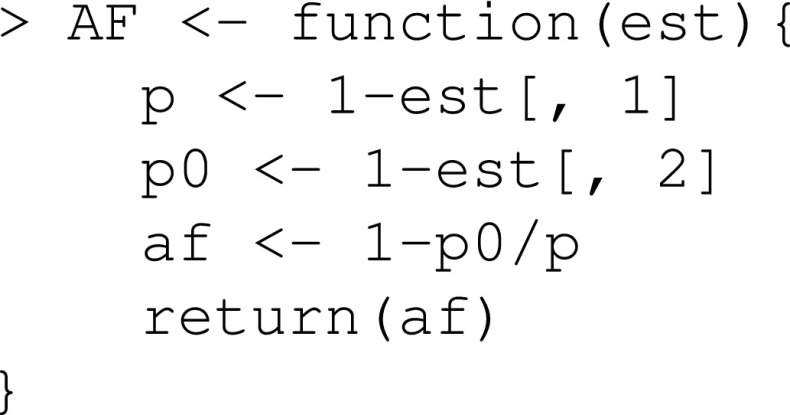


We use this function, and obtain point-wise 95% confidence intervals, by 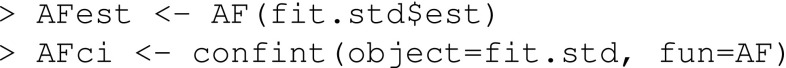


We plot the estimated AF and the confidence intervals by 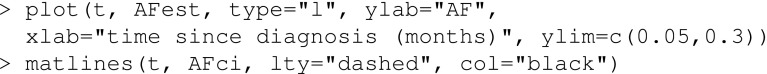


**Fig. 1:**
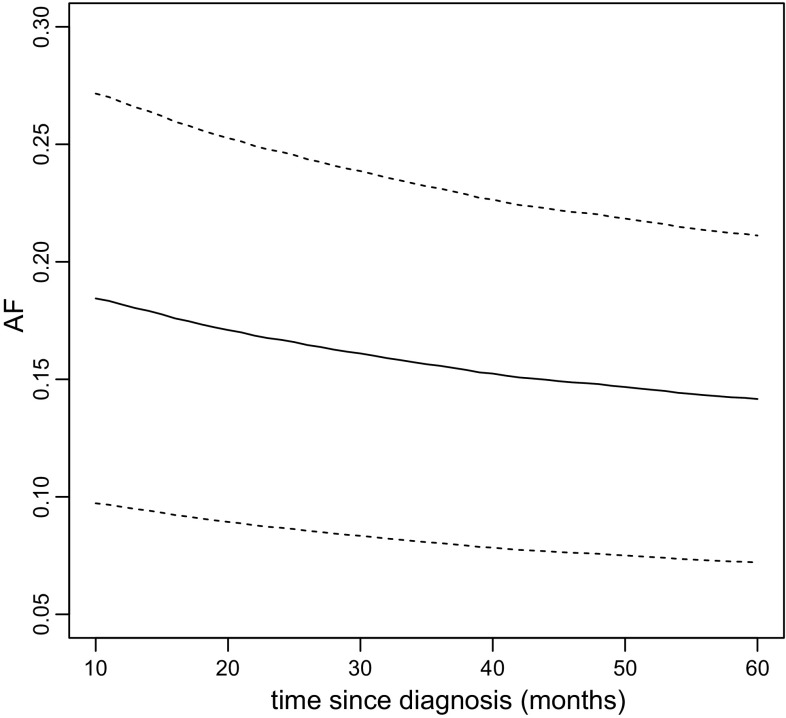
The estimated AF (solid line) as a function of time since diagnosis, together with point-wise 95% confidence intervals (dashed lines)

The resulting plot is displayed in Fig. 1. We observe that the AF declines with time, from 18.4% to 14.2%, at 10 and 60 months after diagnosis, respectively. Thus, the analysis suggests that 18.4% of all deaths/relapses that occurred before 10 months after diagnosis would have been prevented if all patients had been given chemotherapy. When considering a time window up to 60 months after diagnosis, only 14.2% would have been prevented. The point-wise 95% confidence intervals exclude the value 0 everywhere in the time range, which means that we have statistically significant (at 5% significance level) evidence for a preventative effect of chemotherapy everywhere in the time range.

## The NNT

### Definition

The NNT is supposed to measure the average number of subjects that would have to be treated, among those that are factually untreated, to prevent one unfavorable outcome event. In the literature, the NNT is usually defined for binary outcomes and binary treatments as

5 $$\begin{aligned} \frac{1}{p(Y=1|X=0)-p(Y=1|X=1)}, \end{aligned}$$

where $p(Y=1|X=0)$ and $p(Y=1|X=1)$ are the probabilities of the outcome among the untreated and treated, respectively [22]. However, this definition implicitly assumes that there is no confounding, and is thus in practice restricted to randomized control trials. In the presence of confounding, $p(Y=1|X=0)$ may very well be much larger than $p(Y=1|X=1)$ even in the absence of a causal treatment effect, thus falsely implying a small NNT.

To derive a causal definition of the NNT, let *N* be a fixed number of untreated subjects. Among these, $Np(Y=1|X=0)$ subjects will on average have the outcome. Suppose now that we would treat all *N* subjects. Under this counterfactual scenario, the probability of the outcome is $p(Y_1=1|X=0)$; that is, the probability of the outcome if everybody would be treated among those that are factually untreated. Thus, among those *N* subjects that are factually untreated, $Np(Y_1=1|X=0)$ subjects would on average have the outcome if all were treated. Setting $Np(Y=1|X=0)-Np(Y_1=1|X=0)=1$ and solving for *N* gives

6 $$\begin{aligned} \text{NNT}=\frac{1}{p(Y=1|X=0)-p(Y_1=1|X=0)}. \end{aligned}$$

In the absence of confounding, the potential outcome $Y_1$ is equally distributed among treated and untreated, so that $p(Y_1=1|X=0)=p(Y_1=1|X=1)=p(Y=1|X=1)$, and the causal definition in (6) simplifies to the usual definition (6) in the literature.

Estimation of the NNT requires a minor deviation from the recipe outlined in “Regression standardization” section. Because the counterfactual probability $p(Y_1=1|X=0)$ only applies to the subset of the population with $X=0$, we have to replace the averages on the right-hand sides of (1) and (2) with the averages among this subset. We thus have that

7 $$\begin{aligned} p(Y_1=1|X=0)=E\{p(Y=1|X=1,Z)|X=0\}, \end{aligned}$$

and

8 $$\begin{aligned} {\hat{p}}(Y_1=1|X=0)=\frac{\sum _{i=1}^n(1-X_i) {\hat{p}}(Y=1|X=1,Z_i)}{\sum _{i=1}^n(1-X_i)}. \end{aligned}$$

For time-to-event outcomes, we define the NNT as in (6), but with *Y* and $Y_1$ replaced with *Y*(*t*) and $Y_1(t)$, respectively. Thus, the NNT measures the average number of subjects that would have had to be treated at baseline ($t=0$), among those that were factually untreated, in order to prevent one unfavorable outcome event before time *t*.

For details on model-based estimation of the NNT we refer to Bender et al. [23] and Laubender and Bender [24].

### Estimation with logistic regression models

We illustrate the methods with the clslowbwt dataset. We define the ‘treatment’ as absence of the smoking during pregnancy. With this definition, the NNT is interpreted as the average number of smokers that would have to refrain from smoking during pregnancy, in order to prevent one low birthweight.

To be consistent with the notation in “Definition” section, where we used values 0 and 1 for untreated and treated, respectively, we first define the treatment variable 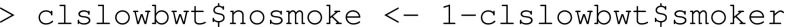


We fit the logistic regression model 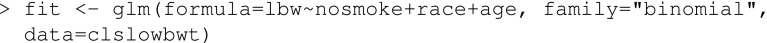


We use the fitted model to estimate standardized probabilities: 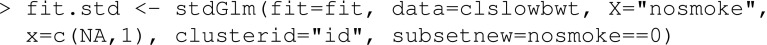


The subsetnew argument specifies a subset of observations to be used when estimating the standardized probabilities. We note that, although we have not used it here, the glm function has a subset argument that allows for subsetting when fitting the regression model. This is different from subsetting when standardizing; thus we have used the term ‘subsetnew’ for the latter. As argued in “Definition” section we only wish to standardize over the untreated (i.e. the smokers) when estimating the NNT; this is achieved by setting subsetnew=nosmoke==0. We note that, by setting x to NA within this subset, we estimate the factual outcome probability among the untreated; $p(Y=1|X=0)$. Summarizing the results gives 
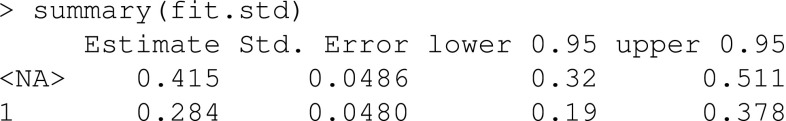


Thus, the factual probability of low birthweight is estimated to be 41.5%, and the counterfactual probability, had nobody smoked, is estimated to be 28.4%. We emphasize that these figures only apply to those who factually did smoke. In contrast, the figures obtained in “Estimation with logistic regression models” section by summary(fit.std) apply to the whole population (i.e. both smokers and non-smokers).

We define a function that estimates the NNT 
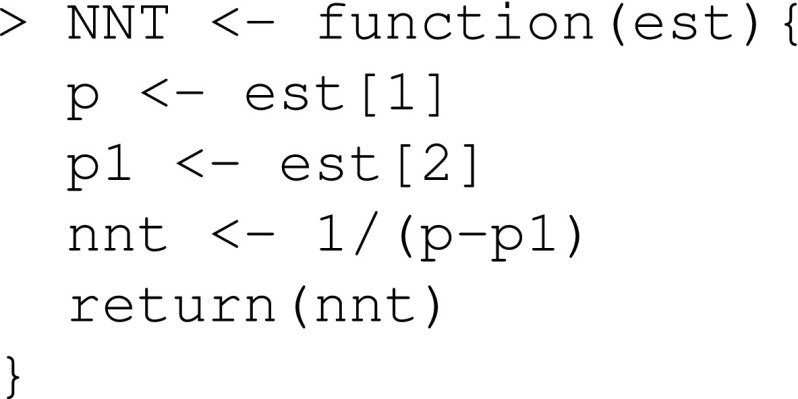


Using the function gives 
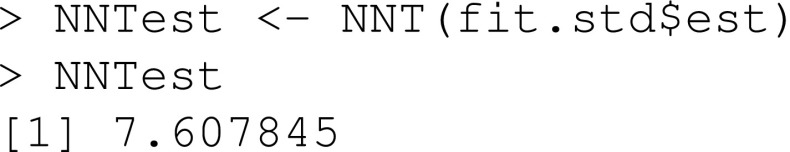


Hence, the analysis suggests that smoking during pregnancy must be prevented for around 7.6 women, in order to prevent one low birthweight. A 95% confidence interval is obtained by 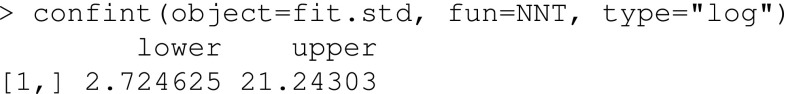


Setting type="log" forces confint to first compute a confidence interval for the logarithm of the NNT, then backtransforming to the original scale. This transformed confidence interval only includes positive values, as it should, but it is quite wide and suggests that the true NNT may be as high as 21.2.

### Estimation with Cox PH regression models

We illustrate the methods with the rott2 dataset. We aim to estimate the average number of patients that would have had to be treated with chemotherapy, among those that were factually untreated, in order to prevent one death/relapse before a specific time-point *t*.

We first fit the Cox PH regression model 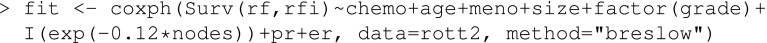


We use the fitted model to estimate standardized probabilities among those that are untreated: 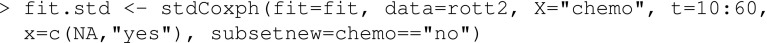


We define a function that estimates the NNT: 
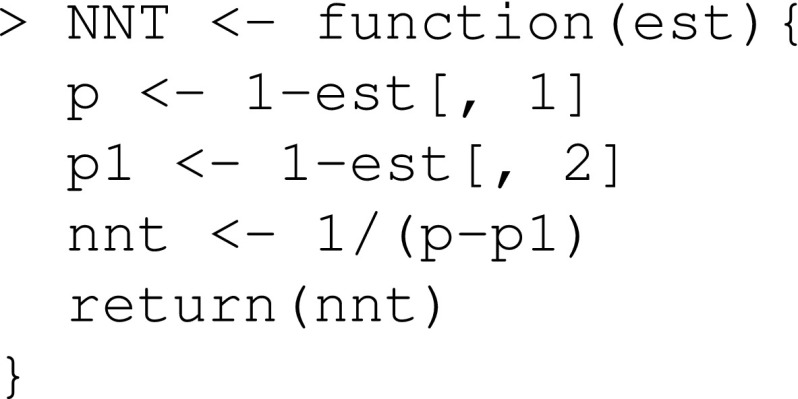


We plot the estimated NNT together with point-wise 95% confidence intervals 
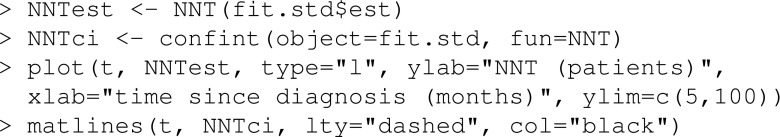


**Fig. 2:**
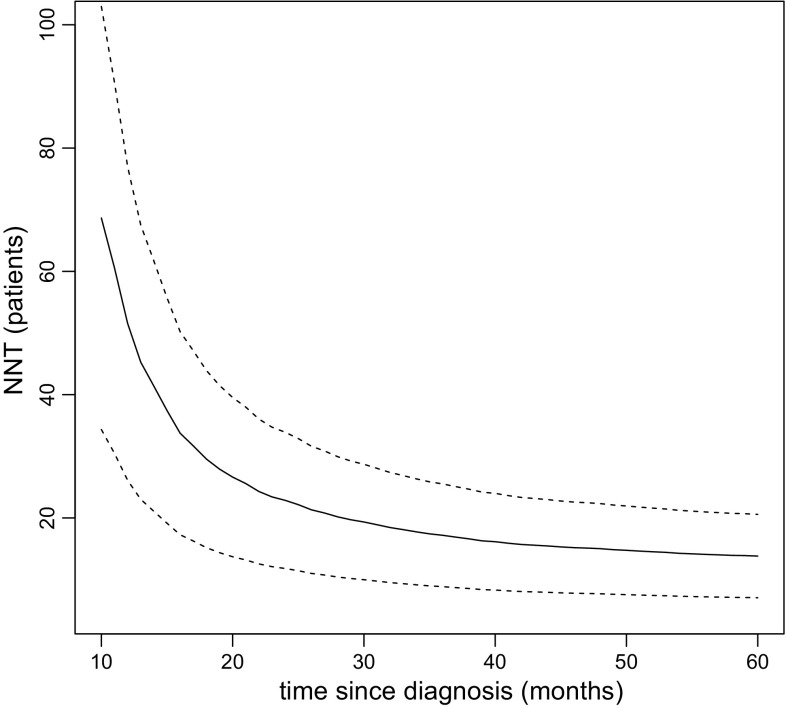
The estimated NNT (solid line) as a function of time since diagnosis, together with point-wise 95% confidence intervals (dashed lines)

The resulting plot is displayed in Fig. 2. We observe that the NNT declines with time, from 68.6 patients to 13.8 patients at 10 and 60 months after diagnosis, respectively. Thus, the analysis suggests that 68.6 patients would have had to be treated, among those that were factually untreated, in order to prevent one death/relapse before 10 months. When considering a time window up to 60 months after diagnosis, it would have been enough to treat 13.8 patients.

## The RERI

### Definition

The RERI is usually defined for two binary exposures. To follow the notation in “Regression standardization” section it is convenient to recode the two exposures into one categorical exposure with levels 00 (both exposures equal to 0), 01 (first exposure equal to 0, second equal to 1), 10 (first exposure equal to 1, second equal to 0) and 11 (both exposures equal to 1). The (causal) RERI is defined as

9 $$\begin{aligned} \text{RERI}=\frac{p(Y_{11}=1)-p(Y_{10}=1)-p(Y_{01}=1) +p(Y_{00}=1)}{p(Y_{00}=1)}, \end{aligned}$$

where, as before, $p(Y_x=1)$ is the counterfactual probability of the outcome if everybody would be exposed to level $X=x$.

The numerator in (9) is the additive interaction between the two exposures. It has been argued that additive interaction is more useful for assessing the public health importance of interventions than interactions on other (e.g. multiplicative) scales. Furthermore, additive interactions can sometimes be used to infer the presence of certain ‘mechanistic/biologic’ interactions (see [4] and the references therein). Because the RERI is defined as the additive interaction divided with the positive constant $p(Y_{00}=1)$, the RERI will always have the same sign (positive, negative or zero) as the additive interaction. We note that there is a wide variety of interaction measures in the epidemiologic literature, and we refer to VanderWeele and Knol [4] for a discussion of their interpretations and relative merits.

For time-to-event outcomes, the RERI is defined as in (9), but with $Y_x$ replaced with $Y_x(t)$ for all *x*. Thus, the RERI becomes a function of time *t*.

### Estimation with logistic regression models

We again illustrate the methods with the clslowbwt dataset, and we let the two exposures of interest be smoker and race. Estimating the causal effect of race poses two important problems. First, it can be argued that the underlying counterfactual query (e.g. ‘what would the probability of the outcome be if everyone was black/white?’) is vague, and that the causal effect of race is thus ill-defined [25]. Second, for any given outcome there is arguably a huge number of risk factors that also correlate with race, and thus the potential for unmeasured confounding is enormous. We ignore these problems here, since our analysis merely serves as an illustration.

To make race binary we restrict the analysis to women who are either black or white, and we define a new four-level exposure as 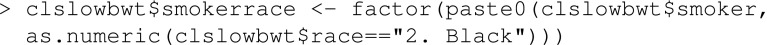


We fit the logistic regression model 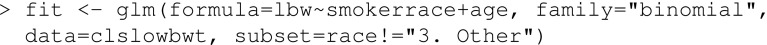


The subsetting on race!="3. Other" restricts the analysis to women who are either black or white. We use the fitted model to estimate standardized probabilities: 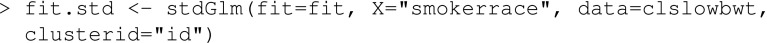


When the exposure is a factor variable, the stdGlm function by default standardize at all exposure levels, which makes it unnecessary to specify the x argument. Summarizing gives 
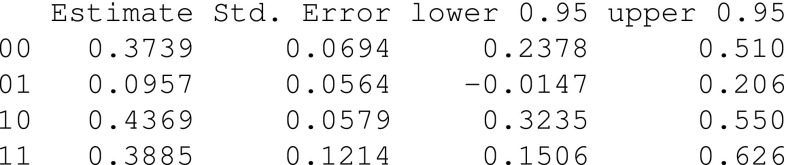


There appears to be a strong heterogeneity in the risk of low birthweight between the four exposure groups. Among black non-smokers ($x=01$) the risk of low birthweight is 9.6%, whereas among white smokers ($x=10$) the risk is 43.7%.

We define a function that estimates the RERI 
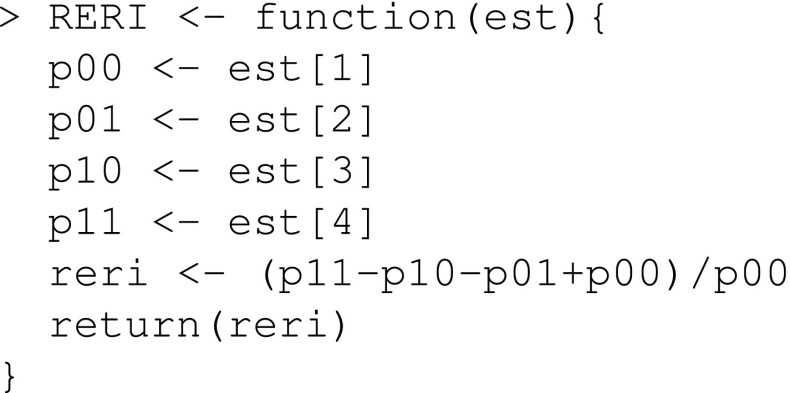


and we use this function to estimate the RERI together with a 95% confidence interval 
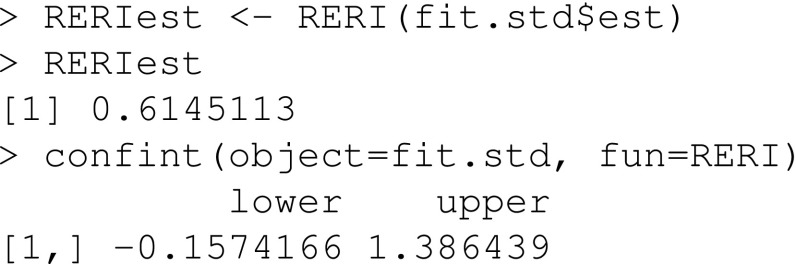


The estimated RERI is equal to 0.61 and the 95% confidence interval includes 0. Thus, we cannot rule out the null hypothesis of no additive interaction between smoking and race.

### Estimation with Cox PH regression models

We again illustrate the methods with the rott2 dataset, and we let the two exposures of interest be nochemo and grade.

We first define a new four-level exposure: 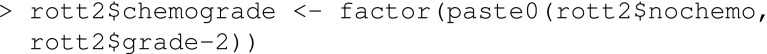


We fit a Cox PH model and use the fitted model to estimate standardized probabilities 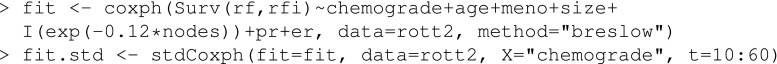


We define a function that estimates the RERI: 
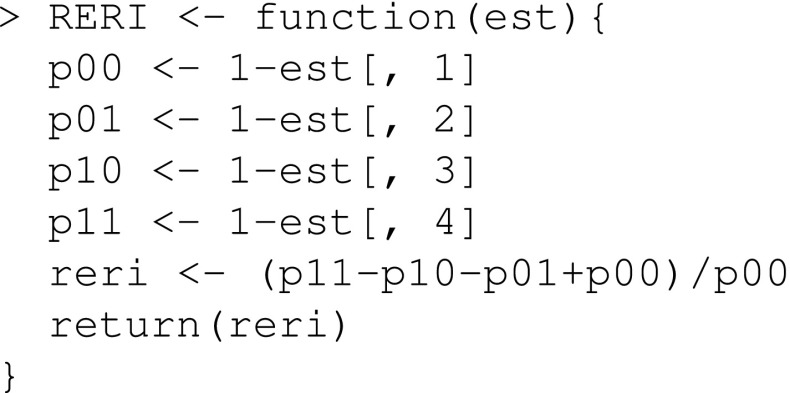


and we use this function to plot the estimated RERI together with point-wise 95% confidence intervals

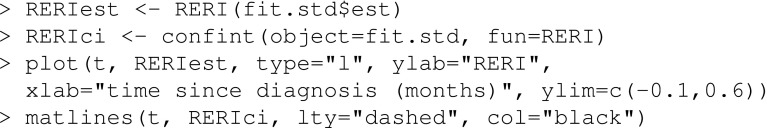



**Fig. 3:**
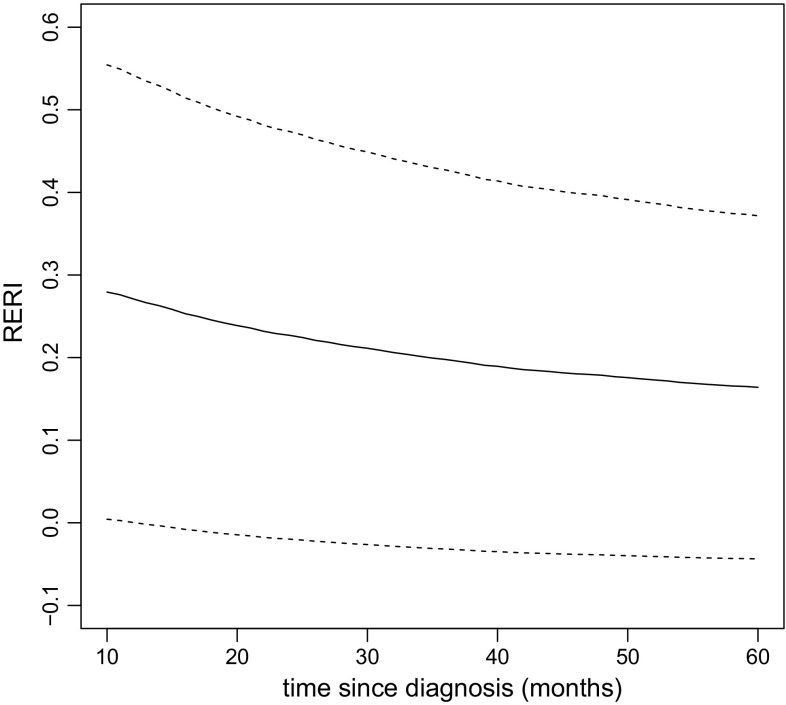
The estimated RERI (solid line) as a function of time since diagnosis, together with point-wise 95% confidence intervals (dashed lines)

The resulting plot is displayed in Fig. 3. We observe that the RERI decreases slightly with time, from 0.28 at 10 months after diagnosis, to 0.16 at 60 months after diagnosis.

## Discussion

Measures of causal effects play a central role in epidemiology. Using appropriate measures when summarizing the results is crucial to make the analysis relevant from a public health perspective. In this paper we have shown how a wide range of effect measures can be estimated with the R-package stdReg, with a minimal effort of programming from the analyst. We have specifically focused on the AF, the NNT and the RERI, but in principle any effect measure can be estimated along the same lines as these, provided that the measure can be written as come contrast between standardized probabilities.

If the confounders included in the regression model are sufficient for confounding control, then standardization estimates the counterfactual probability of the outcome, had everybody in the population attained a fixed level of the exposure. In this sense, standardization estimates population (or marginal) causal effects. An alternative is to use the fitted regression model to estimate causal effects at specific levels of the confounders, i.e. subpopulation (or conditional) causal effects. In the standard use of logistic regression and Cox PH regression it is assumed that the odds ratio and hazard ratio, respectively, are constant across levels of the confounders. However, these models generally imply that other measures, such as the AF and the NNT, vary across confounder levels. To present conditional causal effects, other than the odds ratio of hazard ratio, the analyst would then typically have to restrict attention to a few selected confounder levels, which makes the results less general than when presenting marginal causal effects.

We emphasize that, although slightly beyond the scope of our paper, careful model selection is crucial for estimation of causal effects, and rather different than model selection for prediction. When the aim is to make predictions, one usually attempts to include variables that are strongly associated with the outcome, regardless of the underlying mechanism. Such variables can be selected by fairly automatized procedures, such as step-wise regression. When the aim is to estimate causal effects, one should attempt to include variables that are confounders for the exposure–outcome relationship. Such variables are often strongly associated with the outcome. However, the reverse does not hold; a variable may be strongly associated with the outcome, yet it is not a confounder, and may lead to substantial bias if included in the regression model [10].

The stdReg package uses a fitted regression model to carry out standardization. In this paper we have focused on logistic regression models and Cox PH regression models, since these are the most common models in epidemiology. More generally though, the function stdGlm can be used to carry out standardization with any type of generalized linear model fitted by the glm function, e.g. linear regression or probit regression, as described by Sjölander [7]. The stdReg package also contains a function for standardization with shared frailty gamma-Weibull models, stdParfrailty, which is described by Dahlqwist et al. [26]. In the future we plan to extend the package even further, to allow for standardization with semiparametric frailty models and generalized linear mixed models.

All code in this paper is available at the HTML version of R’s online documentation, which is accessed by help.start().
